# Predicting Smoking Cessation Stages: An Insight from the Transtheoretical Model Using a Cross-Sectional Approach Among Adults in Saudi Arabia

**DOI:** 10.3390/healthcare12232343

**Published:** 2024-11-23

**Authors:** Samiha Hamdi Sayed, Olfat Abdulgafoor Gushgari, Wafaa Taha Ibrahim Elgzar

**Affiliations:** 1 Public Health Department, College of Health Sciences, Saudi Electronic University, Riyadh 11673, Saudi Arabia; 2 Department of Maternity and Childhood Nursing, Nursing College, Najran University, Najran 61441, Saudi Arabia; 3 Department of Obstetrics and Gynecology Nursing, Faculty of Nursing, Damanhour University, Damanhour 22516, Egypt

**Keywords:** adults, smoking, transtheoretical model, Saudi Arabia

## Abstract

Background: Smoking is a detrimental health behavior that can be addressed by designing stage-matched interventions with evidence-based behavioral change models such as the transtheoretical model (TTM). This study applied the TTM to predict smoking cessation stages among adults in Saudi Arabia. Methods: This social media-based cross-sectional study in Saudi Arabia used a convenient sample of 491 adult smokers (men and women). A digital questionnaire containing basic and smoking-related data and smoking scales (stages of change, cessation readiness, decisional balance, and self-efficacy) was used for data collection. The data were collected from 1 July to 30 October 2023 and were investigated using an ordinal regression analysis. The results illustrate that among the studied smokers, cigarette smoking was the prevalent method of smoking, especially among men (71.7%) compared to women (27.8%). Regarding stages of smoking, the pre-contemplation (35.8%) and contemplation (30.1%) stages were the highest, where men were more represented in both stages (37.9% and 40.8%, respectively). In comparison, women represented a higher percentage in the action (23.9%) and maintenance (21.1%) stages. The ordinal regression showed that increasing age (adjusted odds ratio; AOR = 1.045, *p* = 0.044), high quitting readiness (AOR = 1.134, *p* < 0.001), self-efficacy (AOR = 1.965, *p* = 0.028), decisional balance (AOR = 1.870, *p* < 0.001), and absence of psychological problems (AOR = 2.047, *p* < 0.001) increased the likelihood of being at higher smoking cessation stages. However, increased smoking duration (AOR = 0.756, *p* = 0.010), male gender (AOR = 0.340, *p* < 0.001), not working (AOR = 0.364, *p* = 0.013), adequate income (AOR = 0.535, *p* = 0.032), no chronic diseases (AOR = 0.430, *p* < 0.001), regular smoking (AOR = 0.052, *p* < 0.001), high smoking dependency (AOR = 0.775, *p* = 0.038), and hookah smoking (AOR = 0.032, *p* < 0.001) decreased the likelihood of being at higher smoking cessation stages. Conclusions: Cigarette smoking is a prevalent problem among Saudi adults, especially men, with the highest percentage of these being at lower smoking cessation stages. Thus, this study recommends the development of stage-matched interventions to facilitate the move towards higher smoking cessation stages through efforts by, and collaboration between, community sectors to face this rising issue.

## 1. Introduction

Tobacco consumption is a mounting epidemic that threatens public health. Evidence demonstrates that all nicotine-containing tobacco products are highly addictive [1]. There are many forms of tobacco consumption. Tobacco smoking is the most widespread method, which refers to the act of burning tobacco and ingesting its smoke, which may be inhaled, as is the case of cigarettes, or ingested via the mouth, as in the case of pipes and cigars. Smokeless tobacco is another form that is less widespread but must not be neglected as it also poses a significant public health problem in 140 countries all over the world, with approximately 300 million consumers. This involves tobacco consumption without burning or smoking, as in the case of chewing tobacco, snus, snuff, and dissolvable tobacco; thus, it is absorbed through the oral mucosa [2,3].

The World Health Organization (WHO) estimated that in 2020, 22.3% of the world’s population used tobacco, which kills about half of its users if they do not quit. The WHO announced that tobacco kills more than eight million yearly, whereas passive smoking is responsible for almost 1.3 million deaths. Low- and middle-income countries account for more than 80% of tobacco users, with a higher burden of tobacco-related morbidities and deaths [1,4]. In Saudi Arabia, the 2019 Global Adult Tobacco Survey (GATS) estimated that 19.8% of adults in Saudi Arabia are current tobacco users; the percentage of men is much higher (30.0%) than that of women (4.0%). The percentage of current tobacco smoking is 17.9%, which is higher in men (27.5%) than in women (3.7%) [5].

Smoking is a proven risk factor for numerous preventable diseases and premature deaths. In Gulf Cooperation Council countries, smoking was responsible for 16.3% of cancer cases (22.8% among males and 2.8% among females); lung cancer was the most frequent cancer. Urinary bladder and colorectal cancer were also frequent cancers among male smokers, while lip, oral cavity, and cervical cancer were the most frequent cancers among female smokers [6]. A secondary analysis of chronic obstructive pulmonary disease data from the Global Burden of Disease (1990 to 2019) found that its prevalence rate among smokers increased by 49% and the incidence rate increased by 43.4%. This trend was higher among older populations and males than among females [7]. A cross-sectional study in Saudi Arabia showed that smoking was one of the most prevalent risk factors for metabolic syndrome in both genders and across different age groups [8]. Smoking also increases the risk of vascular diseases, strokes, depressed immune function, rheumatoid arthritis, reduced fertility, and erectile dysfunction. Therefore, smoking can drain the country’s resources by direct cost of consumption or through associated work absences and the cost of healthcare services [9,10].

Despite the evidence-based detrimental health impacts of smoking, numerous studies have demonstrated a prevailing lack of knowledge about smoking and its health hazards in the Saudi population [11,12]. Many studies also revealed an independent association between smoking behavior and many sociodemographic factors [13,14]. GATS also reported that about two-fifths of the current smokers in Saudi Arabia initiated smoking quit attempts in the last year [5]. In addition, a recent study proved that 57.0% of the population in Jeddah acknowledged the need to quit [15]. These findings highlight that help is needed by smokers in Saudi Arabia to quit, which necessitates an understanding of the factors associated with quitting behavior. One well-known model that may help with this issue is the transtheoretical model (TTM) [16,17].

The TTM integrates key concepts from multiple behavior change theories and has been extensively validated. It is progressively being used to facilitate change for numerous behaviors, including drug addiction, unhealthy diet, managing stress, and losing weight. It initially proved its efficacy in smoking cessation, where it was broadly acknowledged and practically employed by many clinicians and scientists in the public health field [18,19,20]. The TTM consists of the following four constructs: the stages of change (individually held thoughts and behaviors concerning the targeted behavior); the processes of change (experiential and behavioral techniques employed by the individual during the progression of the stages); self-efficacy to fight the smoking desire; and decisional balance (perception of the cons and pros of the behavior) [21,22].

The TTM assumes that the change in health behavior entails an individual’s progression through five stages, as follows: the pre-contemplation or pre-thinking stage (not intending to quit smoking soon/within six months); the contemplation or thinking stage (planning to quit smoking within six months); the preparation/readiness stage (planning to quit within one month); the action stage (quit smoking for the last six months); and the maintenance stage (keep quitting smoking for six months or more) [21,22]. The TTM operates via the self-efficacy theory, which clarifies its relationship with behavioral change [23]. It demonstrates the importance of an individual’s confidence level for resisting behavior temptation and preventing relapse. In the early stages, temptation tends to be higher than self-confidence; however, in the action stage, both tend to be roughly equal to facilitate behavior change. Hence, the confidence–temptation balance should be maintained as it determines self-efficacy, which is necessary at all stages to prevent relapse [24,25].

During this change process, a person often moves back and forth through stages; many situational, emotional, social, and environmental factors can impede their progress. Recent evidence indicates that marital status, having family members who smoke, educational status, number of quitting attempts, self-efficacy, and quitting readiness play significant roles in individuals’ transition through the smoking stages [26,27,28,29]. Accordingly, exploring the transition stage and its influencing factors will guide the development of stage-directed interventions, which will help to prevent the spiral pattern or return to previous stages [16,17,30]. Moreover, smoking cessation clinics in Saudi Arabia reported a high rate of relapse among consumers of health services. They highlighted the need to give more attention to anti-smoking services to ensure an optimum level of performance [31]. A study in Jazan also explored the need for a more comprehensive smoking cessation intervention package with regular follow-ups to enhance the quit rate [32]. A recent cross-sectional study using the 2019 GAT survey in Saudi Arabia reported a higher awareness and utilization rate of smoking cessation clinics among smokers with higher educational statuses and those living in the urban areas of Riyadh, Makkah, and the Eastern Province of Saudi Arabia. Alternatively, lower awareness was noted among private, self-employed, and unemployed smokers. Knowledge of these factors will help policymakers to dedicate efforts to support those who desire to quit smoking and provide affordable and accessible smoking cessation services [33]. Furthermore, the desire for smoking cessation was positively associated with an awareness of smoking cessation clinics, raising tobacco taxes, and strict home rules against smoking [34].

Hence, the present study can aid in developing targeted educational and smoking control programs. It can also assist healthcare system managers, policymakers, and primary healthcare providers in designing and executing theory-based interventions and appropriate regulations to control smoking in Saudi Arabia. Thus, the aim of this study was to use TTM to predict smoking cessation stages among adult men and women. The study has three primary research objectives, as follows: to investigate the smoking patterns among adult men and women, to explore the smoking behavior stages according to the TTM, and to identify predictors of smoking cessation stage statuses.

## 2. Materials and Methods

### 2.1. Study Design and Setting: A Social Media-Based Cross-Sectional Study

#### 2.1.1. Study Participants and Sampling

The target group was adult men and women in Saudi Arabia (≥20 years). They were incorporated using specific inclusion criteria: being regular or occasional smokers for more than six months before the study, including many forms of smoking (cigarettes, hookah, e-cigarettes, e-hookah), and being willing to participate in the study. The researchers benefited from the features of Survey Monkey software (SurveyMonkey Inc., San Mateo, CA, USA) to specify the participants’ search criteria (all regions of Saudi Arabia, adult age, both genders, and all available social media platforms such as Twitter, LinkedIn, Instagram, Facebook, and Snapchat). In addition, screening questions about smoking status and duration were used at the beginning of the survey to apply the eligibility criteria (if not applicable, the survey was declined). Moreover, many rounds were made to ensure that the survey was distributed to all regions of Saudi Arabia.

The convenience sampling technique was adopted to select participants to fulfill the required sample size, which was determined using the following parameters: Z_α/2_ is 1.96 for alpha 0.05, and P is the proportion of adult smokers in Saudi Arabia, which is (19.8%) based on the last GAT survey; D is the design effect (2) and E is the precision (or margin of error) (0.05) [35]. The resulting sample size was 488; the final number included in the study was 491 smokers.

#### 2.1.2. Instrument Development

The researchers designed a digital structured survey using pertinent and credible studies in the literature. The survey contained the following sections.

##### Basic Personal Data and Health History

Data included, as follows: age in years; gender (male or female); marital status (single or married); nationality (Saudi or non-Saudi); educational level (basic, secondary, university, postgraduate education); residence (central, northern, southern, eastern, western regions); income adequacy (inadequate, adequate, adequate and save); working status (working or not working); and self-reported psychological problems or chronic diseases. (yes/no questions were used to indicate health problems).

##### Smoking Behavior-Related Data

Smoking behavior-related data included, as follows: smoking initiation time, duration, preferred type, perceived dependency, causes of smoking, a smoking family member, quitting trial, and receiving previous medical advice for quitting smoking [27].

##### The Smoking Stages of Change Scale

The Smoking Stages of Change Scale is a staging algorithm based on TTM that was developed by Prochaska et al. (1988); it contains five statements to assess the stages of smoking cessation. The participants nominate one statement that best represents their current smoking behavior change stages: “pre-contemplation” (did not desire to quit smoking in the following six months); “contemplation” (desired to quit smoking in the following six months); “preparation” (plan to quit smoking within the next month with at least one 24 h quitting attempt in the previous year); “action” (did not smoke for less than six months); and “maintenance” (sustained quitting smoking for six months or more) [22,36,37].

##### The Smoking Cessation Readiness Scale

The Smoking Cessation Readiness Scale was adapted from Maryland’s Tobacco Resource Center, United States. It holds seven items rated on a ten-grade readiness ruler, with three readiness levels, where two items have reversed scores. The overall readiness score (7–70) is categorized as low (≤28), moderate (29–49), or high (50–70) [38].

##### Decisional Balance Scale

The Decisional Balance Scale has six items assessing the perceived pros (3 items) and cons (3 items) of smoking. The participants rated every item’s importance according to a 5-point Likert scale from not important (1) to extremely important (5). The scores of the con items were reverse-coded (1 = 5, 2 = 4, 3 = 3, 4 = 2, 5 = 1). The total score (6–30) is categorized as positive (23–30), neutral (15–22), or negative (≤14) [20].

##### Smoking Self-Efficacy Scale

The Smoking Self-Efficacy Scale has nine items related to smoking desire, including, as follows: the individual ability to resist the influence of a negative affect (3 items); social or positive mood (3 items); and craving (3 items). It is rated on a five-point Likert scale ranging from not confident (1) to extremely confident (5). The total score (9–45) is categorized as low (≤21), moderate (22–33), or high (34–45) [39].

### 2.2. Instrument Validity and Reliability

The researchers translated the scales into Arabic using the DeepL Translator software ((DeepL SE Co., Cologne, NW, Germany). An expert researcher conducted a back translation to ensure its accuracy. A panel of six experts in public health reviewed and agreed upon the content of the total instrument by investigating the items’ wording, order, and scoring. Then, it was reviewed and modified according to their feedback, indicating an acceptable Content Validity Index (>0.70). The instrument’s reliability was evaluated by Cronbach’s alpha coefficient test (α) and was proven to have acceptable levels for scales (stages = 0.81, readiness = 0.76, decisional balance = 0.80, self-efficacy = 0.78). 

### 2.3. Pilot Study

The instrument was piloted with 10% of the overall sample size; this was omitted from the main study sample to ensure clarity, wording, and applicability. Accordingly, the desired modifications were performed.

### 2.4. Statistical Analysis

The Survey Monkey program (SurveyMonkey Inc., San Mateo, CA, USA) was used to create and distribute the study’s survey. After gaining ethical approval, the researchers distributed the link to the digital survey using the major social media platforms in Saudi Arabia (Twitter, Instagram, and WhatsApp). It was available from 1 July to 30 October 2023. The participants filled in the survey after answering a filter question about being a smoker. The estimated average survey completion time was 8–10 min, with an 87.0% response rate.

The researchers used IBM Statistical software, version 27 (I.B.M. Corp., Armonk, NY, USA). Descriptive statistics were used to summarize categorical and numerical variables. The normality of the data was assured using the Shapiro–Wilk test (*p* > 0.05). The significance of the statistical differences between men and women was judged using chi-squared or Fisher’s exact tests for categorical variables. The participants’ characteristics, according to the stages of change, were investigated using Cramer’s V test to determine the strength of the relationship between nominal variables of more than two categories. The effect size was categorized as weak (<0.2), moderate (0.2–0.6), and strong (>0.6) [40].

The predictors of smoking cessation stage status were evaluated by logistic ordinal regression using the polytomous universal model (PLUM) and proportional odds model (POM). The authors set the dependent or outcome variable as the staging algorithm of the five stages of smoking behavior change (from pre-contemplation to maintenance) and the independent or predictor variables as personal, health, smoking behavior-related data, decisional balance, cessation readiness, and self-efficacy. The model fitness was confirmed by the likelihood ratio chi-squared test using the (−2 log-likelihood) function; the goodness-of-fit was assured using Pearson’s chi-squared test; and the Nagelkerke test was used to assess the coefficient of determination (Pseudo R2) of the model. The ordered log-odds (logit) regression coefficients were used to determine the estimates of the predictors. The parallel lines test confirmed the assumption of proportional odds. The cut-off value of significance was *p* < 0.05 [41,42].

### 2.5. Ethical Considerations

Ethical approval was obtained from the institutional review board of the Saudi Electronic University on 18 June 2023 (SEUREC-4457). After explaining the study’s aim and importance, informed digital consent was obtained initially and individually from each respondent. The researchers told the respondents that their data were anonymous. They were also assured that data would be kept confidential and deployed only to serve the scientific research objectives. The respondents were also informed of their right to withdraw at any time.

## 3. Results

### 3.1. Participants’ Personal, Health, and Smoking-Related Data

Among the 491 participants, 311 (63.3%) were men and 180 (36.7%) were women, with a higher mean age among men (30.46) than women (27.36). The highest percentage of men (63.0%, 71.1%) and women (70.6%, 67.8%) were married and had a university education, respectively. Most men (76.5%) were working, 58.9% of women were students, and most were Saudis (93.6% of men and 94.4% of women). Residence in the central region was noted among 32.8% of men compared to 20.8% of women and the highest percentage of men (47.9%) and women (53.7%) perceived an adequate income. Most men (79.0%) and women (75.0%) had no chronic diseases, while 66.1% of women reported having no psychological problems compared to 46.6% of men. Statistically significant differences were detected across all variables (*p* < 0.05) except for nationality, marital status, and chronic diseases (*p* > 0.05) [Table 1].

Table 2 illustrates that cigarette smoking was the highest among men (71.7%) compared to women (27.8%). Duration of smoking was higher among men at 57.6% (11–15 years) compared to women at 24.4%. Daily regular smoking was reported by 75.9% of men compared to 45.0% of women, and 29.6% of men perceived a very high smoking dependency compared to 6.7% of women. Stress was the chief perceived cause of smoking among men (46.9%) and women (43.3%). Most men (70.4%, 79.1%) and women (84.4%, 74.4%) had a smoking family member and a quitting trial, respectively. However, 34.4% of men received medical advice to quit smoking compared to 16.1% of women. Statistically significant differences were noted across all variables (*p* < 0.05) except for the quitting trial (*p* > 0.05).

### 3.2. Participants’ Smoking Stage and Its Relationship with Their Characteristics and Smoking Behavior

**Figure 1:**
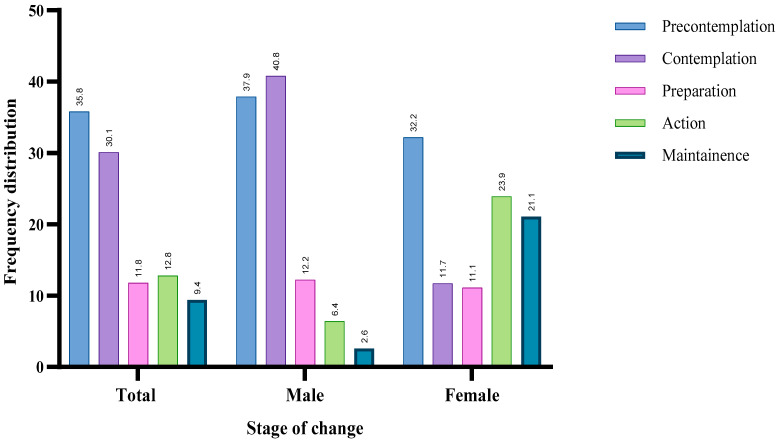
Frequency distribution of the participants by their smoking cessation stage and gender.

Around one-third of the studied participants were either at the pre-contemplation (35.8%) or contemplation (30.1) stages. Men represented the majority for both stages (37.9%, 40.8%) compared to 32.2% and 11.7% of women, respectively. Conversely, a minority were either at the action (12.8%) or maintenance (9.4%) stages, whereas women represented a higher percentage for both stages (23.9% and 21.1%, respectively) [Figure 1].

Table 3 portrays significant moderate relationships between smoking stages and regularity of smoking (φ*_c_* = 0.474), gender (φ*_c_* = 0.456), quitting readiness (φ*_c_* = 0.434), decisional balance (φ*_c_* = 0.406), self-efficacy (φ*_c_* = 0.377), psychological issues (φ*_c_* = 0.371), smoking dependency (φ*_c_* = 0.285), and smoking cause (φ*_c_* = 0.280) (*p* < 0.001). Weak significant relationships were noted for the absence of chronic diseases (φ*_c_* = 0.248), favorite smoking method (φ*_c_* = 0.221), marital status (φ*_c_* = 0.213), working status (φ*_c_* = 0.210), education (φ*_c_* = 0.168) (*p* < 0.001), age (φ*_c_* = 0.167, *p* = 0.018), income adequacy (φ*_c_* = 0.165, *p* < 0.001), medical advice to quit (φ*_c_* = 0.145, *p* = 0.034), and residence (φ*_c_* = 0.123, *p* = 0.019). However, having smoking family members (*p* = 0.092) and nationality (*p* = 0.194) had insignificant relationships with smoking stages.

### 3.3. Predictors of Smoking Cessation Across the Five Stages of Change Using Ordinal Regression Model

The ordinal regression model shows significance in predicting smoking stage status (χ^2^ = 483.13, −2 log likelihood = 957.31, *p* = 0.000) and Pearson goodness of fit (χ^2^ = 2028.84, *p* = 0.878), and the pseudo-R^2^ (Nagelkerke = 0.661), with an insignificant test of parallel lines (χ^2^ = 123.01, −2 log likelihood = 902.11, *p* = 0.360). The model shows that increasing age (AOR = 1.045; 95%CI = 1.001, 1.090; *p* = 0.044), quitting readiness (AOR = 1.134; 95%CI = 1.108–1.160; *p* < 0.001), self-efficacy (AOR = 1.965; 95%CI = 1.935–1.996; *p* = 0.028), decisional balance (AOR = 1.870; 95%CI = 1.818–1.926; *p* < 0.001), and having no psychological problems (AOR = 2.047; 95%CI = 1.343–3.119; *p* < 0.001) increased the likelihood of being at higher smoking cessation stages [Table 4].

Alternatively, increased smoking duration (AOR = 0.756; 95%CI = 0.611–0.936; *p* = 0.010), being men (AOR = 0.340; 95%CI = 0.188–0.615; *p* < 0.001), not working (AOR = 0.364; 95%CI = 0.164–0.808; *p* = 0.013), living in the eastern (AOR = 0.492; 95%CI = 0.247–0.982; *p* = 0.044) and western (AOR = 0.282; 95%CI = 0.134–0.595; *p* < 0.001) regions of Saudi Arabia, perceived adequate income (AOR = 0.535; 95%CI = 0.302–0.949; *p* = 0.032), absence of chronic diseases (AOR = 0.430; 95%CI = 0.269–0.686; *p* < 0.001), regularity of smoking (AOR = 0.052; 95%CI = 0.024–0.114; *p* < 0.001), perceived high smoking dependency (AOR = 0.775; 95%CI = 0.471–0.791; *p* = 0.038), and hookah smoking (AOR = 0.032; 95%CI = 0.008–0.133; *p* < 0.001) decreased the likelihood of being at higher smoking cessation stages [Table 4].

## 4. Discussion

This study showed that cigarette smoking was the prevalent method of smoking among about three-quarters of men. At the same time, more than a quarter of women smoked either cigarettes or hookah, and more than one-third used e-cigarettes. However, e-hookah was minimally used. Stress was the chief perceived cause of smoking, and most men and women had no chronic diseases. However, a higher percentage of men reported psychological issues than women; stress, anxiety, tension, and depressed mood were the most frequently reported issues. These findings show that cigarette smoking is widespread in Saudi Arabia, especially among adult men, but the findings are hopeful because most men and women had previous smoking quitting trials.

Recent evidence supports the current study findings; smoking is the most common form of tobacco use not only in Saudi Arabia [34,43] but also in Indonesia [44], Iran [45], and South Asia [46]. Evidence also shows that most smokers had no comorbidities [43,44,45], a high smoking dependency, and a low starting age [43]. Moreover, a study in South Asia by Timilsina et al. (2022) revealed that mental stress was the main reason for the early initiation of smoking. However, it showed that smoking dependency was high in men and women, which is not in line with the present study, where men had significantly higher dependency levels than women [46]. These conflicting results may be due to the smaller sample size in this conflicting study (280), with fewer female participants (61).

This study found that approximately two-thirds of the participants were in the pre-contemplation and contemplation stages, where men represented most of these early stages. However, women represented most of the action and maintenance stages. These findings were supported by a significant positive relationship between smoking stages and gender, where men’s gender was shown to decrease the likelihood of being in the higher stages of smoking cessation. These findings could be explained by the higher smoking dependency, psychological problems, and regular daily smoking among men than women, which showed moderate significant relationships with the stages of smoking. These findings shed light on the significant actions required to motivate smokers and boost their readiness for quitting smoking.

Similarly, a study by Moqaddas et al. (2023) revealed that most smokers were in the pre-contemplation and contemplation stages, while a minority were in other stages [26]. An Iranian study by Emadzadeh and Vakili (2020) revealed that most smoking males were in the pre-contemplation stage, with a significant difference between men and women [45]. Conversely, an Indonesian study by Permana et al. (2021) showed that about half of the smokers were in the preparation stage, while around one-quarter were in the pre-contemplation or contemplation stage [44]. This conflict in the stages of change in Permana’s study compared to the present study could be attributed to the small sample size (150) and the conduction of the study among HIV patients who were obligated to initiate smoking quitting due to their immunodeficient status.

The present study revealed that increasing age, quitting readiness, self-efficacy, and decisional balance with no psychological problems increased the likelihood of being in the higher stages of smoking cessation. These could be explained by the tendency of self-reevaluation and initiating action to quit smoking while getting older, which is enforced by a higher readiness, self-efficacy, and the perceived benefits of smoking cessation with the psychological ability to do so. Alternatively, increased smoking duration, male gender, unemployment, living in the eastern and western regions of Saudi Arabia., perceived adequate income, absence of chronic diseases, regularity of smoking, perceived high dependency, and hookah smoking decreased the likelihood of being in the higher stages of smoking cessation. These findings highlight the complexity of interweaving factors hindering smoking cessation decisions, which necessitate immediate action.

Consistent findings in a study by Moqaddas et al. (2023) showed that higher age and economic status, being married, and having a disease were significantly related to the stages of change [26]. A Chinese study by Wang et al. (2023) found that low willpower, tobacco dependence, bad moods, and work- or life-related stress were among the adverse factors for quitting smoking failure [47]. A study in Zambia by Zyambo et al. (2023) showed that older age and having mental health problems were significantly associated with higher odds of smoking. Conversely, being female and diabetic decreased the odds of tobacco smoking [48]. Timilsina et al. (2022) explored that a significantly higher percentage of smokers in the early stages of change had a high smoking dependency [46]. Emadzadeh and Vakili (2020) found that having a male gender and hookah smoking were predictors of being in the pre-contemplation stage among Iranians [45]. A study in Vietnam by Luu et al. (2020) indicated that women had a higher success rate of quitting smoking than men. It also showed that living in a context where smoking is an acceptable societal norm, with frequent exposure to pro-tobacco advertising coupled with social isolation, might decrease the chances of successful quitting [49]. This could explain the present study’s findings, which demonstrate that living in the eastern and western regions of Saudi Arabia increased the likelihood of being at lower smoking stages.

A Chinese study by He et al. (2020) illustrated that smokers who had extended tobacco consumption durations had greater difficulty in quitting smoking due to higher dependency levels. It also explored associations between smoking dose, duration, and poor health status, indicating that having a chronic disease, regardless of having a shorter or longer smoking duration, decreased the likelihood of smoking cessation [50]. Wang et al. (2019) added that despite the prevalence of longer smoking durations, smokers with lower smoking intensities had a higher likelihood of smoking cessation [51]. A Korean study by Leem et al. (2017) added that those with a sufficient income were more likely to be in the pre-contemplation stage [52]. Latifi et al. (2017) showed that self-efficacy was significantly higher at the maintenance stage and lowest at the pre-contemplation stage [29]. Moreover, an Iranian study by Narimani et al. (2017) showed that decisional balance and quitting readiness were significantly correlated with positive progress in the smoking stages [28]. All these consistent findings illustrate the multifactorial nature of smoking behavior and cessation decisions, highlighting the need for collaborative, individual, community, government, and policy actions to cut down this rising phenomenon.

Many studies show contrasting findings. Emadzadeh and Vakili (2020) showed that the ages of smokers were higher at the pre-contemplation and contemplation stages than at other stages. This could be explained by stage variations in this conflicting study, where most participants were at the termination stage; in addition, smokers in the study had a lower mean age than smokers in the present study [45]. A study from Lebanon by Bacha et al. (2018) showed that smokers with high nicotine dependence had greater success quitting smoking [53]. These conflicting findings might be explained by the smaller sample size in Bacha’s study and the variation in the measurement method of dependency; in the current study, dependency was self-reported, while in Bacha’s study, it was objectively measured using urinary cotinine samples.

Moreover, in Vietnam, Luu et al. (2020) found that unemployed smokers had a higher probability of succeeding in quitting smoking, which the authors suggested was illogical, as tobacco is now prohibited in numerous workplaces. This conflict may be explained by variations in the participants’ employment status; in the present study, the highest percentage of participants were employed, while in Luu’s study, most were unemployed. Thus, we can suggest that being unemployed might increase stress, compromising an individual’s ability to quit smoking. Luu et al. (2020) also showed that being married and highly educated increases the likelihood of quitting smoking [49], while Moqaddas et al. (2023) found that the higher the educational level, the higher the odds of being in the lower stages of change [26]. Although the present study detected weak significant relationships between marital status and education with the smoking stages, they were not significant predictors of moving through the stages. This conflict could be explained by variations in participants’ characteristics; these two conflicting studies had a higher percentage of single participants with lower educational statuses compared to the high rate of married participants with bachelor’s degrees in the present study. To sum up, variations and inconsistencies in smoking-associated factors demonstrates that smoking is a sociocultural concern that varies across societies, and its predictors vary as a result of complex and multi-faceted issues.

### Strengths, Limitations, and Future Implications

The current study used a robust design to study the smoking cessation stages using the TTM among adult male and female smokers in Saudi Arabia. It also examined the factors that influence quitting decisions using a theory-based framework for analysis. The study was cautious in its representation of the five geographical regions of Saudi Arabia. However, the study has many, perhaps inevitable limitations, including the social desirability bias linked to self-reported data, where respondents may give answers that they believe are socially desirable rather than their actual opinions or experiences. In addition, using social media platforms for data collection introduces the potential for selection bias among social media users.

Hence, the current study recommends the development of quitting smoking hotlines to offer support for smokers; designing capacity-building media campaigns to enhance awareness about smoking’s negative consequences; and establishing effective counseling service packages, based on the TTM, at smoking cessation clinics to encourage quitting. A further longitudinal follow-up study to examine smoking cessation stage transitions is also recommended.

## 5. Conclusions

The present study showed that cigarette smoking was the prevalent method of smoking, especially among adult men. The highest proportion of smokers were in the pre-contemplation and contemplation stages; men were overrepresented for these early stages, whereas women represented most of the later stages (action and maintenance) of smoking cessation. Increasing age, quitting readiness, self-efficacy, decisional balance, and having no psychological problems were significant predictors of smoking cessation. However, increased smoking duration, being men, unemployed, living in the eastern and western regions of Saudi Arabia, perceived adequate income, absence of chronic diseases, regularity of smoking, perceived high smoking dependency, and hookah smoking were barriers to smoking cessation decisions. Thus, smoking is an alarming issue in Saudi Arabia. Aside from the high rate, especially among men, most of the smokers were in the early stages of change, which highlights the need for further actions to support these groups.

## Figures and Tables

**Table 1:** Participants’ basic and health-related data.

Parameters	Categories	Men *n* (311)	Women *n* (180)	Test (*p* Value)
		No. (%)	No. (%)	
Age (years)	21–30	181 (58.2)	133 (73.9)	t = 4.717 (<0.001 *)
	31–40	102 (32.8)	35 (19.4)	
	41–50	28 (9.0)	12 (6.7)	
	Mean ± SD (95%CI)	30.46 ± 6.72 (29.71–31.21)	27.36 ± 7.54 (26.25–28.46)	
Marital status	Single	115 (37.0)	53 (29.4)	χ^2^ = 2.874 (0.090)
	Married	196 (63.0)	127 (70.6)	
Education	Basic	10 (3.2)	0 (0.0)	χ^2^ = 10.463 (0.015 *)
	Secondary	60 (19.3)	36 (20.0)	
	University	221 (71.1)	122 (67.8)	
	Postgraduate	20 (6.4)	22 (12.2)	
Working status	Working	238 (76.5)	42 (23.3)	χ^2^ = 131.686 (<0.001 *)
	Not working	16 (5.1)	32 (17.8)	
	Student	57 (18.4)	106 (58.9)	
Nationality	Saudi	291 (93.6)	170 (94.4)	χ^2^ = 0.152 (0.696)
	Non-Saudi	20 (6.4)	10 (5.6)	
Residence region	Central	102 (32.8)	37 (20.6)	χ^2^ = 19.934 (<0.001 *)
	Eastern	73 (23.5)	55 (30.6)	
	Western	61 (19.6)	52 (28.9)	
	Northern	43 (13.8)	11 (6.1)	
	Southern	32 (10.3)	25 (13.9)	
Perceived income adequacy	Inadequate	106 (34.1)	67 (37.2)	χ^2^ = 7.569 (0.002 *)
	Adequate	149 (47.9)	97 (53.9)	
	Adequate and save	56 (18.0)	16 (8.9)	
Chronic diseases	No	246 (79.0)	135 (75.0)	FET = 1.102 (0.294)
	Respiratory	13 (4.2)	15 (8.4)	
	Cardiovascular	14 (4.5)	2 (1.1)	
	Obesity	12 (3.9)	6 (3.3)	
	Diabetes	10 (3.2)	10 (5.6)	
	Stress colon	12 (3.9)	4 (2.2)	
	Anemia	4 (1.3)	8 (4.4)	
Psychological problems	No	143 (46.0)	119 (66.1)	FET = 18.564 (<0.001 *)
	Anxiety	57 (18.3)	27 (15.0)	
	Tension	35 (11.3)	10 (5.6)	
	Depressed mood	16 (5.1)	4 (2.2)	
	Stress	60 (19.3)	20 (11.1)	

t: independent sample *t* test; χ^2^: chi-squared test; FET: Fisher’s exact test; * Significant at *p* ≤ 0.05.

**Table 2:** Participants’ smoking-related data.

Parameters	Categories	Men *n* (311)	Women *n* (180)	Test (*p* Value)
		No. (%)	No. (%)	
Favorite smoking method	Cigarettes	223 (71.7)	50 (27.8)	FET = 97.64 (<0.001 **)
	Hookah	46 (14.8)	50 (27.8)	
	e-cigarettes	40 (12.9)	68 (37.8)	
	e-hookah	2 (0.6)	12 (6.7)	
Duration of smoking (years)	<1	3 (1.0)	30 (16.7)	FET = 80.48 (<0.001 **)
	1–5	80 (25.7)	74 (41.1)	
	6–10	49 (15.8)	32 (17.8)	
	11–15	179 (57.6)	44 (24.4)	
Regularity of smoking	Regular (Daily)	236 (75.9)	81 (45.0)	χ^2^ = 70.30 (<0.001 **)
	Regular (Weekly)	38 (12.2)	20 (11.1)	
	Irregular	37 (11.9)	79 (43.9)	
Perceived smoking dependency	No	47 (15.1)	64 (35.6)	χ^2^ = 57.51 (<0.001 **)
	Low	30 (9.6)	28 (15.6)	
	Moderate	72 (23.2)	27 (15.0)	
	High	70 (22.5)	49 (27.2)	
	Very high	92 (29.6)	12 (6.7)	
Perceived smoking causes	Stress	146 (46.9)	78 (43.3)	χ^2^ = 10.45 (0.015 *)
	Peer pressure	26 (8.4)	32 (17.8)	
	Curiosity	74 (23.8)	33 (18.3)	
	Admiration	65 (20.9)	37 (20.6)	
Having a smoking family member	Yes	219 (70.4)	152 (84.4)	χ^2^ = 12.15 (<0.001 **)
	No	92 (29.6)	28 (15.6)	
Medical advice to quit	Yes	107 (34.4)	29 (16.1)	χ^2^ = 19.05 (<0.001 **)
	No	204 (65.6)	151 (83.9)	
Previous quitting trial	Yes	246 (79.1)	129 (74.4)	χ^2^ = 1.41 (0.235)
	No	65 (20.9)	46 (25.6)	

χ^2^: chi-squared test; FET: Fisher’s exact test; * Significant at *p* ≤ 0.05; ** Significant at *p* ≤ 0.01.

**Table 3:** The participants’ characteristics and smoking behavior by the stage of change.

Variables	Categories	Total n (%)	P.C. n (%)	C n (%)	P n (%)	A n (%)	M n (%)	φ*_c_* *(p* Value)
Age (years)	21–30	314 (64.0)	98 (31.2)	100 (31.8)	39 (12.4)	45 (14.3)	32 (10.2)	0.167 (0.018)
	31–40	137 (27.9)	62 (45.3)	40 (29.2)	15 (10.9)	10 (7.3)	10 (7.3)	
	41–50	40 (8.1)	16 (40.0)	8 (20.0)	4 (10.0)	8 (20.0)	4 (10.0)	
Gender	Male	311 (63.3)	118 (37.9)	127 (40.8)	38 (12.2)	20 (6.4)	8 (2.6)	0.456 (<0.001 **)
	Female	180 (36.7)	58 (32.2)	21 (11.7)	20 (11.1)	43 (23.9)	38 (21.1)	
Marital status	Single	168 (34.2)	80 (47.6)	44 (26.2)	10 (6.0)	24 (14.3)	10 (6.0)	0.213 (<0.001 **)
	Married	323 (65.8)	96 (29.7)	104 (32.2)	48 (14.9)	39 (12.1)	36 (11.1)	
Education	Basic	10 (2.0)	2 (20.0)	8 (80.0)	0 (0.0)	0 (0.0)	0 (0.0)	0.168 (<0.001 **)
	Secondary	96 (19.6)	27 (28.1)	34 (35.4)	11 (11.5)	8 (8.3)	16 (16.7)	
	University	343 (69.9)	131 (38.2)	98 (28.6)	47 (13.7)	45 (13.1)	22 (6.4)	
	Postgraduate	42 (8.6)	16 (38.1)	8 (19.0)	0 (0.0)	10 (23.8)	8 (19.0)	
Working status	Working	280 (57.0)	106 (37.9)	97 (34.6)	35 (12.5)	28 (10.0)	14 (5.0)	0.210 (<0.001 **)
	Not working	48 (9.8)	14 (29.2)	10 (20.8)	0 (0.0)	16 (33.3)	8 (16.7)	
	Student	163 (33.2)	56 (34.4)	41 (25.2)	23 (14.1)	19 (11.7)	24 (14.7)	
Nationality	Saudi	461 (93.9)	166 (36.0)	136 (29.5)	56 (12.1)	57 (12.4)	46 (10.0)	0.111 (0.194)
	Non-Saudi	30 (6.1)	10 (33.3)	12 (40.0)	2 (6.7)	6 (20.0)	0 (0.0)	
Residence region	Central	129 (28.3)	50 (36.0)	43 (30.9)	23 (16.5)	9 (6.5)	14 (10.1)	0.123 (0.019 *)
	Eastern	128 (26.1)	42 (32.8)	40 (31.2)	12 (9.4)	24 (18.8)	10 (7.8)	
	Western	113 (23.0)	47 (41.6)	35 (31.0)	6 (5.3)	19 (16.8)	6 (5.3)	
	Northern	54 (11.0)	21 (38.9)	15 (27.8)	9 (16.7)	3 (5.6)	6 (11.1)	
	Southern	57 (11.6)	16 (28.1)	15 (26.3)	8 (14.0)	8 (14.0)	10 (17.5)	
Perceived income adequacy	Inadequate	173 (35.2)	65 (37.6)	53 (30.6)	23 (13.3)	8 (4.6)	24 (13.9)	0.165 (<0.001 **)
	Adequate	246 (50.1)	87 (35.4)	69 (28.0)	29 (11.8)	47 (19.1)	14 (5.7)	
	Adequate and save	72 (14.7)	24 (33.3)	26 (36.1)	6 (8.3)	8 (11.1)	8 (11.1)	
Chronic diseases	No	381 (77.6)	158 (41.5)	113 (29.7)	37 (9.7)	45 (11.8)	28 (7.3)	0.248 (<0.001 **)
	Yes	110 (22.4)	18 (16.4)	35 (31.8)	21 (19.1)	18 (16.4)	18 (16.4)	
Psychological problems	No	262 (53.4)	91 (34.7)	60 (22.9)	18 (6.9)	51 (19.5)	42 (16.0)	0.371 (<0.001 **)
	Yes	229 (46.6)	85 (37.1)	88 (38.4)	40 (17.5)	12 (5.2)	4 (1.7)	
Self-efficacy	Low	105 (21.4)	44 (41.9)	26 (24.8)	18 (17.1)	9 (8.6)	8 (7.6)	0.377 (<0.001 **)
	Moderate	239 (48.7)	92 (38.5)	72 (30.1)	35 (14.6)	16 (6.7)	24 (10.0)	
	High	147 (29.9)	28 (19.0)	33 (22.4)	35 (23.8)	37 (25.2)	14 (9.5)	
Decisional balance	Negative	112 (22.8)	63 (56.2)	19 (17.0)	16 (14.3)	8 (7.1)	6 (5.4)	0.406 (<0.001 **)
	Neutral	310 (63.1)	113 (36.5)	120 (38.7)	34 (11.0)	25 (8.1)	18 (5.8)	
	Positive	69 (14.1)	0 (0.0)	9 (13.0)	8 (11.6)	30 (43.5)	22 (31.9)	
Quitting readiness	Low	56 (11.4)	47 (83.9)	3 (5.4)	0 (0.0)	0 (0.0)	6 (10.7)	0.434 (<0.001 **)
	Moderate	266 (54.2)	113 (42.5)	105 (39.5)	13 (4.9)	23 (8.6)	12 (4.5)	
	High	169 (34.4)	16 (9.5)	40 (23.7)	45 (26.6)	40 (23.7)	28 (16.6)	
Smoking family member	No	120 (24.4)	50 (41.7)	40 (33.3)	14 (11.7)	8 (6.7)	8 (6.7)	0.127 (0.092)
	Yes	371 (75.6)	126 (34.0)	108 (29.1)	44 (11.9)	55 (14.8)	38 (10.2)	
Medical advice to quit	No	372 (72.3)	133 (37.5)	103 (29.0)	48 (13.5)	45 (12.7)	26 (7.3)	0.145 (0.034 *)
	Yes	136 (27.7)	43 (31.6)	45 (33.1)	10 (7.4)	18 (13.2)	20 (14.7)	
Perceived smoking cause	Stress	224 (45.6)	88 (39.3)	79 (35.3)	14 (6.2)	35 (15.6)	8 (3.6)	0.280 (<0.001 **)
	Peer pressure	58 (11.8)	7 (12.1)	21 (36.2)	0 (0.0)	22 (37.9)	8 (13.8)	
	Curiosity	107 (21.8)	37 (34.6)	24 (22.4)	22 (20.6)	2 (1.9)	22 (20.6)	
	Admiration	102 (20.8)	44 (43.1)	24 (23.5)	22 (21.6)	4 (3.9)	8 (7.8)	
Smoking duration	<1	33 (6.7)	3 (9.1)	0 (0.0)	0 (0.0)	18 (54.5)	12 (36.4)	0.306 (<0.001 **)
	1–5	154 (31.4)	46 (29.9)	53 (34.4)	32 (20.8)	9 (5.8)	14 (9.1)	
	6–10	81 (16.5)	38 (46.9)	12 (14.8)	15 (18.5)	10 (12.3)	6 (7.4)	
	11–15	223 (45.4)	89 (39.9)	83 (37.2)	11 (4.9)	26 (11.7)	14 (6.3)	
Favorite smoking method	Cigarette	273 (55.6)	100 (36.6)	93 (34.1)	34 (12.5)	18 (6.6)	28 (10.3)	0.221 (<0.001 **)
	Hookah	96 (19.6)	46 (47.9)	24 (25.0)	16 (16.7)	6 (6.2)	4 (4.2)	
	e-cigarettes	108 (22.0)	26 (24.1)	31 (28.7)	6 (5.6)	33 (30.6)	12 (11.1)	
	e-hookah	14 (2.9)	4 (28.6)	0 (0.0)	2 (14.3)	6 (42.9)	2 (14.3)	
Regularity of smoking	Regular (Daily)	315 (64.2)	151 (47.9)	116 (36.8)	32 (10.2)	10 (3.2)	6 (1.9)	0.474 (<0.001 **)
	Regular (Weekly)	58 (11.8)	16 (27.6)	10 (17.2)	8 (13.8)	4 (6.9)	20 (34.5)	
	Irregular	118 (24.0)	9 (7.6)	22 (18.6)	18 (15.3)	49 (41.5)	20 (16.9)	
Perceived smoking dependency	No	111 (22.6)	25 (22.5)	18 (16.2)	24 (21.6)	16 (14.4)	28 (25.2)	0.285 (<0.001 **)
	Low	58 (11.8)	12 (20.7)	4 (6.9)	8 (13.8)	22 (37.9)	12 (20.7)	
	Moderate	99 (20.2)	36 (36.4)	42 (42.4)	10 (10.1)	11 (11.1)	0 (0.0)	
	High	119 (24.2)	59 (49.6)	35 (29.4)	9 (7.6)	10 (8.4)	6 (5.0)	
	Very high	104 (21.2)	44 (42.3)	49 (47.1)	7 (6.7)	4 (3.8)	0 (0.0)	

φ*_c_* = Cramer’s V test, * Significant at *p* ≤ 0.05, ** Significant at *p* ≤ 0.01, P.C. = precontemplation, C = contemplation, P = preparation, A = action, M = maintenance.

**Table 4:** Ordinal regression analysis of the predictors of smoking cessation across the five stages of change.

Variables	Categories	Estimate	S.E.	Wald	EXP_B (A.O.R.)	0.95% CI		*p* Value
						LL	UL	
Age	(Years)	0.044	0.022	4.069	1.045	1.001	1.090	0.044 *
Initiation age	(Years)	0.121	0.027	19.494	0.886	0.839	0.935	0.675
Smoking duration	(Years)	−0.279	0.109	6.571	0.756	0.611	0.936	0.010 *
Self-efficacy	Total score	0.036	0. 016	4.859	1.965	1. 935	1. 996	0.028 *
Decisional balance	Total score	0.139	0. 032	19.380	1.870	1.818	1.926	<0.001 **
Quitting readiness	Total score	0.125	0.012	116.380	1.134	1.108	1.160	<0.001 **
Gender	Men	−1.078	0.302	12.760	0.340	0. 188	0.615	<0.001 **
	Women	-	-	-	1	-	-	-
Nationality	Saudi	0.673	0.424	2.519	1.960	0.854	4.501	0.113
	Non-Saudi	-	-	-	1	-	-	-
Marital status	Single	0.053	0.260	0.041	1.054	0.633	1.756	0.840
	Married	-	-	-	1	-	-	-
Education	Basic	−1.044	0.791	1.741	0.352	0.075	1.660	0.187
	Secondary	−0.720	0.459	2.463	0.487	0.198	1.196	0.117
	University	−0.679	0.407	2.782	0.507	0.228	1.126	0.095
	Postgraduate	-	-	-	1	-	-	-
Working status	Working	−0.221	0.285	0.602	0.802	0.459	1.401	0.438
	Not working	−1.010	0.407	6.173	0.364	0.164	0.808	0.013 *
	Student	-	-	-	1	-	-	-
Nationality	Saudi	0.673	0.424	2.519	1.960	0.854	4.501	0.113
	Non-Saudi	-	-	-	1	-	-	-
Residence region	Central	−0.623	0.345	3.270	0.536	0.273	1.054	0.071
	Eastern	−0.709	0.352	4.049	0.492	0.247	0.982	0.044 *
	Western	−1.265	0.381	11.034	0.282	0.134	0.595	<0.001 **
	Northern	−0.679	0.411	2.730	0.507	0.227	1.135	0.098
	Southern	-	-	-	1	-	-	-
Perceived income level	Inadequate	−0.382	0.307	1.556	0.682	0.374	1.244	0.212
	Adequate	−0.626	0.292	4.580	0.535	0.302	0.949	0.032 *
	Adequate and save	-	-	-	1	-	-	-
Chronic diseases	No	−0.844	0.239	12.516	0.430	0.269	0.686	<0.001 **
	Yes	-	-	-	1	-	-	-
Psychological problems	No	0.716	0.215	11.092	2.047	1.343	3.119	<0.001 **
	Yes	-	-	-	1	-	-	-
Regularity of smoking	Regular (Daily)	−2.949	0.395	55.801	0.052	0.024	0.114	<0.001 **
	Regular (Weekly)	0.116	0.363	0.102	1.123	0.551	2.287	0.749
	Irregular	-	-	-	1	-	-	-
Perceived smoking dependency	No	0.499	0.460	1.178	1.647	0.669	4.059	0.278
	Low	0.047	0.442	0.011	0.954	0.401	2.268	0.915
	Moderate	0.187	0.356	0.276	0.829	0.412	1.667	0.599
	High	−0.386	0.327	1.394	0.775	0.471	0.791	0.038 *
	Very high	-	-	-	1	-	-	-
Favorite smoking method	Cigarettes	−0.163	0.694	0.055	0.850	0.218	3.311	0.815
	Hookah	−3.428	0.720	22.638	0.032	0.008	0.133	<0.001 **
	e-cigarettes	−0.375	0.702	0.286	0.687	0.174	2.720	0.593
	e-hookah	-	-	-	1	-	-	-

SE: standard error, AOR: adjusted odds ratio, CI: 95% confidence interval for AOR, L.L.: lower limit of 95% CI, U.L.: upper limit of 95% CI, Exp_B: Exponential Estimate, * Significant at *p* ≤ 0.05. ** Significant at *p* ≤0.001.

## Data Availability

Data can be obtained from the corresponding author.
